# Memory-based optical polarization conversion in a double-$\Lambda$ atomic system with degenerate Zeeman states

**DOI:** 10.1038/s41598-020-70810-8

**Published:** 2020-08-19

**Authors:** Yan-Cheng Wei, Sheng-Xiang Lin, Pin-Ju Tsai, Ying-Cheng Chen

**Affiliations:** 1 Institute of Atomic and Molecular Sciences, Academia Sinica, Taipei, 10617 Taiwan; 2 Department of Physics, National Taiwan University, Taipei, 10617 Taiwan; 3 Center for Quantum Technology, Hsinchu, 30013 Taiwan

**Keywords:** Optics and photonics, Physics

## Abstract

Optical memory based on the electromagnetically induced transparency (EIT) in a double-$\Lambda$ atomic system provides a convenient way to convert the frequency, bandwidth or polarization of an optical pulse by storing it in one $\Lambda$ channel and retrieving it from another. This memory-based optical converter can be used to bridge the quantum nodes which have different physical properties in a quantum network. However, in real atoms, each energy level usually contains degenerate Zeeman states, which may lead to additional energy loss, as has been discussed in our recent theoretical paper (Tsai et al. in Phys. Rev. A 100, 063843). Here, we present an experimental study on the efficiency variation in the EIT-memory-based optical polarization conversion in cold cesium atoms under Zeeman-state optical pumping. The experimental results support the theoretical predictions. Our study provides quantitative knowledge and physical insight useful for practical implementation of an EIT-memory-based optical converter.

## Introduction

The storage and retrieval of light pulses in atomic ensembles using the effect of electromagnetically induced transparency (EIT) in a three-level $\Lambda$-system has been intensively studied. It has the important application to implement optical quantum memory for quantum information processing^1,2^. By controlling the intensity, frequency or direction of the control field during the retrieval process, the temporal width, frequency or propagating direction of the retrieved probe pulses can be manipulated^3–6^. With a four-level double-$\Lambda$ system, the wavelength of the retrieved probe pulses can be widely manipulated by turning on the control field of the second $\Lambda$ system during the retrieval process^6–9^. This can serve as a quantum frequency converter for the interface between different quantum nodes in a quantum network. Furthermore, by turning on both control fields during retrieval, the stored atomic coherence can be simultaneously released into two separate photonic channels with the amplitude ratio controlled by the intensity ratio of the two control fields^5,7,8,10^, thereby serving as a frequency-domain tunable beam splitter^11^ or two-color quantum memory^12^.

However, each energy level usually contains degenerate Zeeman states in real atoms, which may induce some complications in the memory-based optical conversion with a double-$\Lambda$ system. In a recent theoretical work^13^, we identified the two factors affecting the efficiency of the converted pulses. The first factor is the finite bandwidth effect of the optical pulses and the difference in the optical depth of the storage and retrieval channels. The second factor is the incompatibility between the stored ground-state coherence and the ratio of the probe and control Clebsch–Gordan coefficients of the conversion channel, which leads to a nonadiabatic energy loss in the retrieved pulses^6,7,14^. We obtained an approximate relation for the conversion efficiency. In addition, we also numerically studied the dependence of those two factors on the Zeeman population distribution, facilitated by optical pumping^13^.

In this paper, we present an experimental study on EIT-memory-based optical polarization conversion in a double-$\Lambda$ system with degenerate Zeeman states, using cold cesium atoms. Specifically, we study the dependence of the conversion efficiency on various configurations, including the Zeeman population distribution prepared by optical pumping, the pulsed or continuous-wave probe, and the difference in optical depth for the storage and retrieval channel. The experimental observations support the experimental predictions. Our work provides useful quantitative knowledge and insight into the phenomena of optical conversion based on EIT-memory.

## Results

### Theoretical model

**Figure 1:**
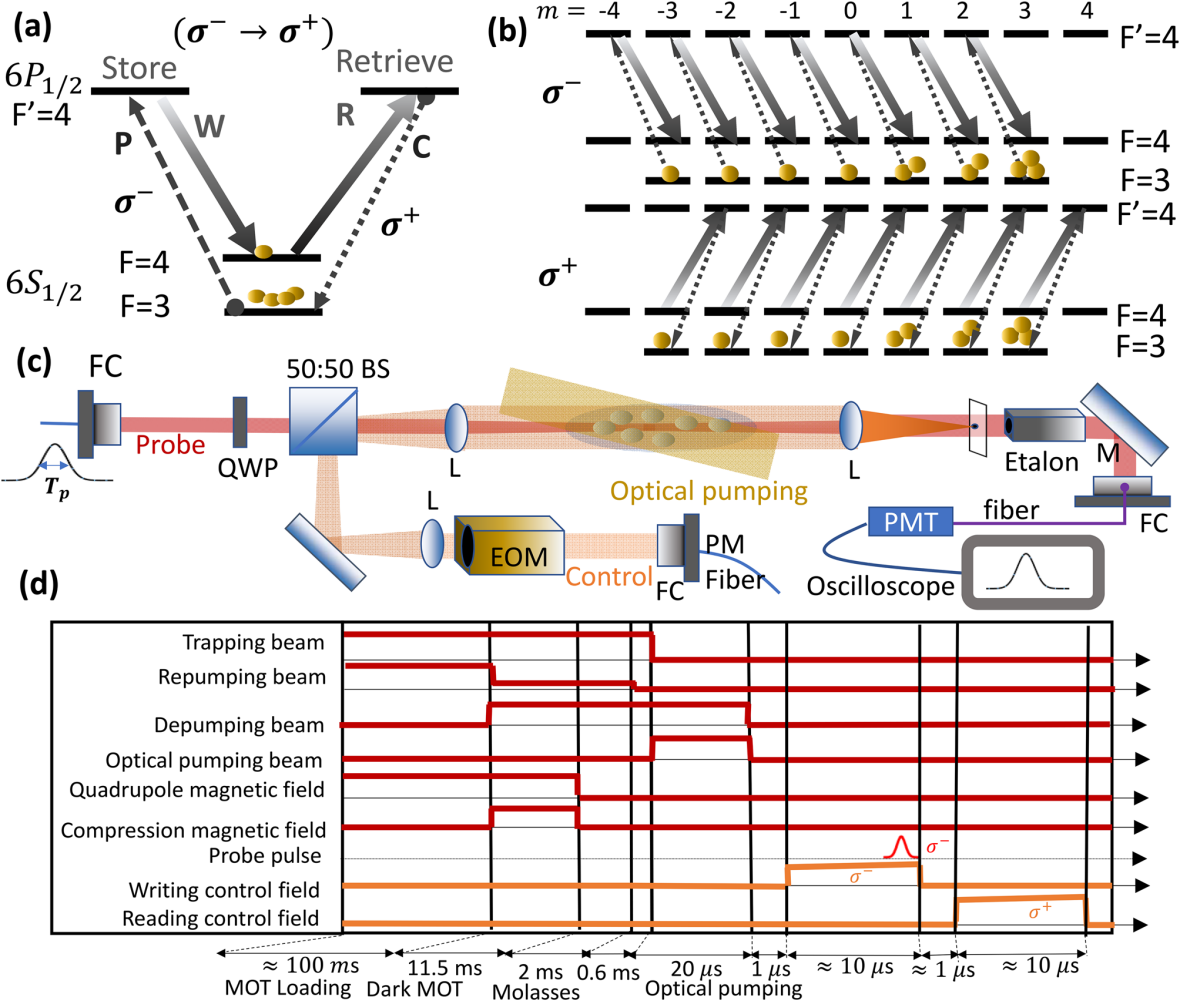
(**a**) Relevant energy levels for $^{133}\hbox {Cs}$ atoms and laser excitations. In this figure, we plot the case where light is converted and stored in the spin-wave with a left-hand circularly polarized beam ($\sigma ^-$ transition) to be retrieved with a right-hand circularly polarized beam ($\sigma ^+$ transition). W, R, P, C denote the writing control, reading control, probe, and conversion fields, respectively. This case and the opposite case, i.e. storing by the $\sigma ^+$ transitions and retrieving by the $\sigma ^-$ transitions, are both implemented in the experiment. (**b**) The complete energy levels and laser excitations considering the degenerate Zeeman states. (**c**) Schematic experimental setup. BS: beam splitter; M: mirror; L: lens; FC: fiber coupler; QWP: quarter waveplate; PMT: photo-multiplier tube; EOM: electro-optic modulator; PM fiber: polarization-maintaining fiber. (**d**) Timing sequence for the polarization conversion experiment.

Before discussing the experiment, we give a brief summary of the theoretical background^13^. Consider EIT-memory-based optical polarization conversion in a double-$\Lambda$ system with degenerate Zeeman states as shown in Fig. 1a and b for the cesium $D_1$ line. In the $\sigma ^- \rightarrow \sigma ^+$ ($\sigma ^+ \rightarrow \sigma ^-$) conversion system, both the probe and writing control beams drive the $\sigma ^-$ ($\sigma ^+$) transitions in the storage channel and the conversion and reading control fields drive the $\sigma ^+$ ($\sigma ^-$) transitions in the retrieval channel. We define a relative conversion efficiency, $\xi ^R_c$, which is the ratio of the energy of the converted pulse to that retrieved in the original storage channel. The absolute conversion efficiency can be simply obtained by the product of the relative conversion efficiency and the storage-and-retrieval efficiency in the original $\Lambda$ system. The storage-and-retrieval efficiency in a single $\Lambda$ system is already well studied, which requires a high optical depth and a low ground-state decoherence rate to achieve a high memory efficiency^15–17^.The $\xi ^R_c$ allows one to focus the discussion on the conversion efficiency dependence due to the different physical properties of the two $\Lambda$ systems during retrieval process. Based on the Maxwell-Bloch equations and under certain approximations, we obtain^13^

1 $$\begin{aligned} \begin{aligned} \xi ^R_c&=\xi _1(\eta ) \ \xi _2, \\ \xi _1(\eta )&= \frac{\zeta _p(\eta )}{\zeta _c(\eta )}, \ \ \xi _2 = \frac{\left| \sum _j p_j R_j^p R_j^c\right| ^2}{\sum _j p_j {R_j^p}^2\sum _j p_j {R_j^c}^2}, \end{aligned} \end{aligned}$$

where $\zeta _p, \zeta _c$ are given by

2 $$\begin{aligned} \begin{aligned} \zeta _p(\eta )&= \left[ 1+\frac{16\text {ln2}(\eta -\kappa )\eta }{\beta ^2_w(L_w)}\frac{\sum _j p_j {R_j^p}^4/(a_{p,j}^2 \alpha _p)}{{\left( \sum _j p_j {R_j^p}^2\right) }^2}\right] ^{\frac{1}{2}}, \\ \zeta _c(\eta )&= \left[ 1+\frac{16\text {ln2}(\eta -\kappa )\eta }{\beta ^2_w(L_w)}\frac{\sum _j p_j {R_j^c}^4/(a_{c,j}^2\alpha _c)}{{\left( \sum _j p_j {R_j^c}^2\right) }^2}\right] ^{\frac{1}{2}} \end{aligned} \end{aligned}$$

and

3 $$\begin{aligned} \begin{aligned} \beta _w(L_w)=\left[ 1 + 16\text {ln2}\eta \kappa \frac{\sum _j p_j {R_j^p}^4/(a_{p,j}^2 \alpha _p)}{{\left( \sum _j p_j {R_j^p}^2\right) }^2}\right] ^{\frac{1}{2}}, \end{aligned} \end{aligned}$$

where $R^p_j= \frac{a_{p,j}}{a_{w,j}}$, $R^c_j = \frac{a_{c,j}}{a_{r,j}}$, $\eta \equiv \frac{T_d}{T_p}$, $\kappa \equiv \frac{T_w}{T_d}$, and $L_w=v_w T_w\simeq T_w L/T_d=L\kappa /\eta$. $v_w$ is the group velocity of the probe pulse in the storage channel. Subscript *j* denotes the *j*th Zeeman sub-level, and $p_j$ denotes the population in the *j*th ground state of the probe transition; $a_{(p,c,r,w)}$ denotes the Clebsch–Gordon coefficients for the probe, conversion, reading control, and writing control transitions, respectively. $\alpha _p$ and $\alpha _c$ are the normalized optical depth of the probe and conversion transition without being multiplied by the Clebsch–Gordan coefficients. $T_d$ denotes the group delay time of the slow light and can be expressed as $\Gamma \sum _j \frac{p_j a_{p,j}^2\alpha _p}{|a_{w,j}^2\Omega _w|^2}$, where $\Omega _w$ is the Rabi frequency of the writing control field without multiplied by the Clebsch–Gordon coefficients and $\Gamma$ denotes the spontaneous decay rate; $T_w$ denotes the time when the writing control beam is switched off; $T_p$ denotes the intensity full width at half maximum (FWHM) duration of the input probe pulse with a Gaussian waveform.

**Figure 2:**
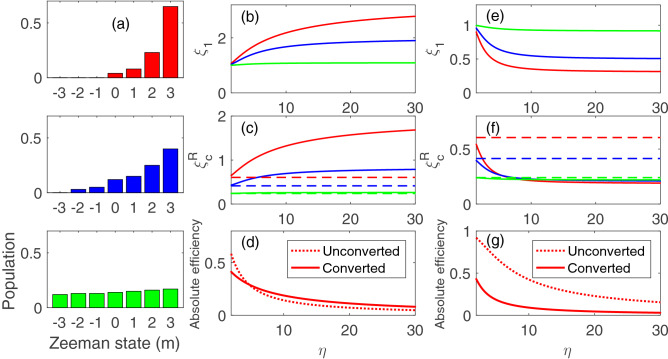
Theoretical calculations based on Eq. (1): (**a**) three assumed Zeeman population distributions. From bottom to top, the Zeeman population changes from a near isotropic distribution to mostly being concentrated in the rightmost Zeeman state. The optical depth for the probe driving the $\sigma ^+$ ($\sigma ^-$) transition increases (decreases) from the bottom population to the top. The solid curves in (**b**–**d**) (**e**–**g**) are $\xi _1$, $\xi _c^R$ and absolute efficiency versus $\eta$ for the $\sigma ^- \rightarrow \sigma ^+$ ($\sigma ^+ \rightarrow \sigma ^-$) conversion system. The color of the curves indicates the corresponding Zeeman population of (**a**) of the same color used in the calculation. The dashed lines in (**c**) and (**f**) are the corresponding $\xi _2$. In (**d**) and (**g**), we plot the absolute efficiency for the unconverted and converted cases for the top (red) Zeeman population distribution. The parameters $\alpha _p$ and $\alpha _c$ are both equal to 500 and $\kappa$ is 1.1.

In Eq. (1), the relative conversion efficiency is determined by the product of two factors, which we term the finite bandwidth factor ($\xi _1$) and the ground-state coherence mismatch factor ($\xi _2$)^13^. $\xi _2$, ranging from zero to unity, stands for a nonadiabatic energy loss during the retrieval process when the population is distributed among several Zeeman states and a mismatch exists on the ratio $R_j^p/R_j^c$ for any of the *j*th sub-system^13,14^. The other factor $\xi _1$ is due to the finite bandwidth effect of the optical pulse and the difference in optical depth between the storage and retrieval channels^13^. In the pulsed probe case, $\xi _1$ depends on the parameter $\eta =T_d/T_p$. Because the EIT bandwidth of the storage channel $\Delta \omega _{EIT}\propto \Omega _w^2/(\sqrt{\alpha _p}\Gamma )$ and the spectral bandwidth of the probe pulse $\Delta \omega _p \propto 1/T_p$, $\eta \propto \sqrt{\alpha _p}\Delta \omega _p/\Delta \omega _{EIT}$, which is related to the ratio of the pulse bandwidth to EIT bandwidth^17^. This is why we call $\xi _1$ the finite-bandwidth factor.

To obtain a clearer picture of the trend based on Eq. (1), examine Fig. 2b–d and e–g which depict the theoretical plots of $\xi _1$, $\xi _c^R$ and absolute efficiency versus $\eta$ for the $\sigma ^- \rightarrow \sigma ^+$ and $\sigma ^+ \rightarrow \sigma ^-$ conversion system, respectively. The three solid curves (red, blue and green) in Fig. 2b,c and e,f correspond to the three Zeeman population distributions of the same colors in Fig. 2 a. As a reference, we also plot the absolute efficiency for the unconverted and converted cases as shown in Fig. 2d,g. For clarity, we only show the case corresponding to the top (red) Zeeman population distribution. The three dashed lines in (c,f) are the corresponding $\xi _2$, which are independent of $\eta$. Some trends are worthy to mention. First, $\xi _1$ is larger (smaller) than unity for the $\sigma ^- \rightarrow \sigma ^+$ ($\sigma ^+ \rightarrow \sigma ^-$) conversion system. As discussed in Ref.^13^, the retrieval efficiency is related to the delay-bandwidth product of the retrieved transition, which is dependent on the optical depth only. The retrieval channel with a larger optical depth has a higher retrieval efficiency. Considering the Clebsch–Gordan coefficients for the cesium $D_1$ transition and the assumed Zeeman population, the $\sigma ^- \rightarrow \sigma ^+$ ($\sigma ^+ \rightarrow \sigma ^-$) conversion system corresponds to the conversion from a channel with a smaller (larger) optical depth to that with a larger (smaller) one. Second, the deviation of $\xi _1$ from unity is larger for the Zeeman population with a more concentrated distribution towards the $|m=3\rangle$ state. This is due to a larger contrast in the Clebsch–Gordan coefficient for the $\sigma ^-$ and $\sigma ^+$ probe transitions for the Zeeman state with a larger *m* quantum number. In the case with an isotropic Zeeman population, the overall optical depths for the $\sigma ^-$ and $\sigma ^+$ probe transitions are equal and thus $\xi _1$ is equal to unity. Third, $\xi _2$ increases with a more concentrated Zeeman distribution and is actually equal to unity when the whole population is in a single Zeeman state^13^. Fourth, although $\xi _1 >1$ for the $\sigma ^- \rightarrow \sigma ^+$ system, $\xi _c^R(=\xi _1\xi _2)$ is larger than unity only if the Zeeman population is concentrated enough and if $\eta$ is larger than a certain value, as shown in Fig. 2c. Even though the relative conversion efficiency is larger than unity, this situation occurs at a relatively large $\eta$ such that the absolutely conversion efficiency is significantly less than unity, as shown in Fig. 2d. Fifth, $\xi _1$ approaches unity when $\eta$ decreases for both $\sigma ^- \rightarrow \sigma ^+$ and $\sigma ^+ \rightarrow \sigma ^-$ conversion systems. A smaller $\eta$ corresponds to a stronger writing control field and thus a wider EIT transparent bandwidth, compared to the spectral bandwidth of the probe pulse. The finite-bandwidth effect is thus less significant.

### Experimental details

Our experiment is based on a cesium magneto-optical trap (MOT) with optically dense cold atomic media. Details of the MOT and experimental setup are shown in Refs.^17,18^. The relevant energy levels and laser excitations are shown in Fig. 1a. For the storage channel, the writing control field and the probe field drive the $$|F=4\rangle \rightarrow |F'=4\rangle$$ and $$|F=3\rangle \rightarrow |F'=4\rangle$$$$\sigma ^{-}$$ transition of the cesium $$D_1$$ line, respectively. For the retrieval channel, the reading control field and the conversion field drive the same transitions but with $$\sigma ^{+}$$ polarizations, as shown in Fig. 1a. We compare this conversion system with that of the opposite driving case, i.e., storing with $$\sigma ^{+}$$ transitions and retrieving with $$\sigma ^{-}$$ transitions. Each hyperfine level in Fig. 1a contains multiple degenerate Zeeman states. The complete laser excitations are shown in Fig.1b. In this experiment, we manipulate the polarization of the retrieved light but not its frequency. Although it is also possible to manipulate the frequency of the retrieved pulses by choosing another control transition (e. g. the $$|F=4\rangle \rightarrow |F'=4\rangle$$ transition of the $$D_2$$ line) during the retrieval process, our scheme allows us to concentrate on the relative efficiency change of the retrieved pulses without being concerned with the systematic effect due to the detection of retrieved pulses of different wavelengths.

**Figure 3:**
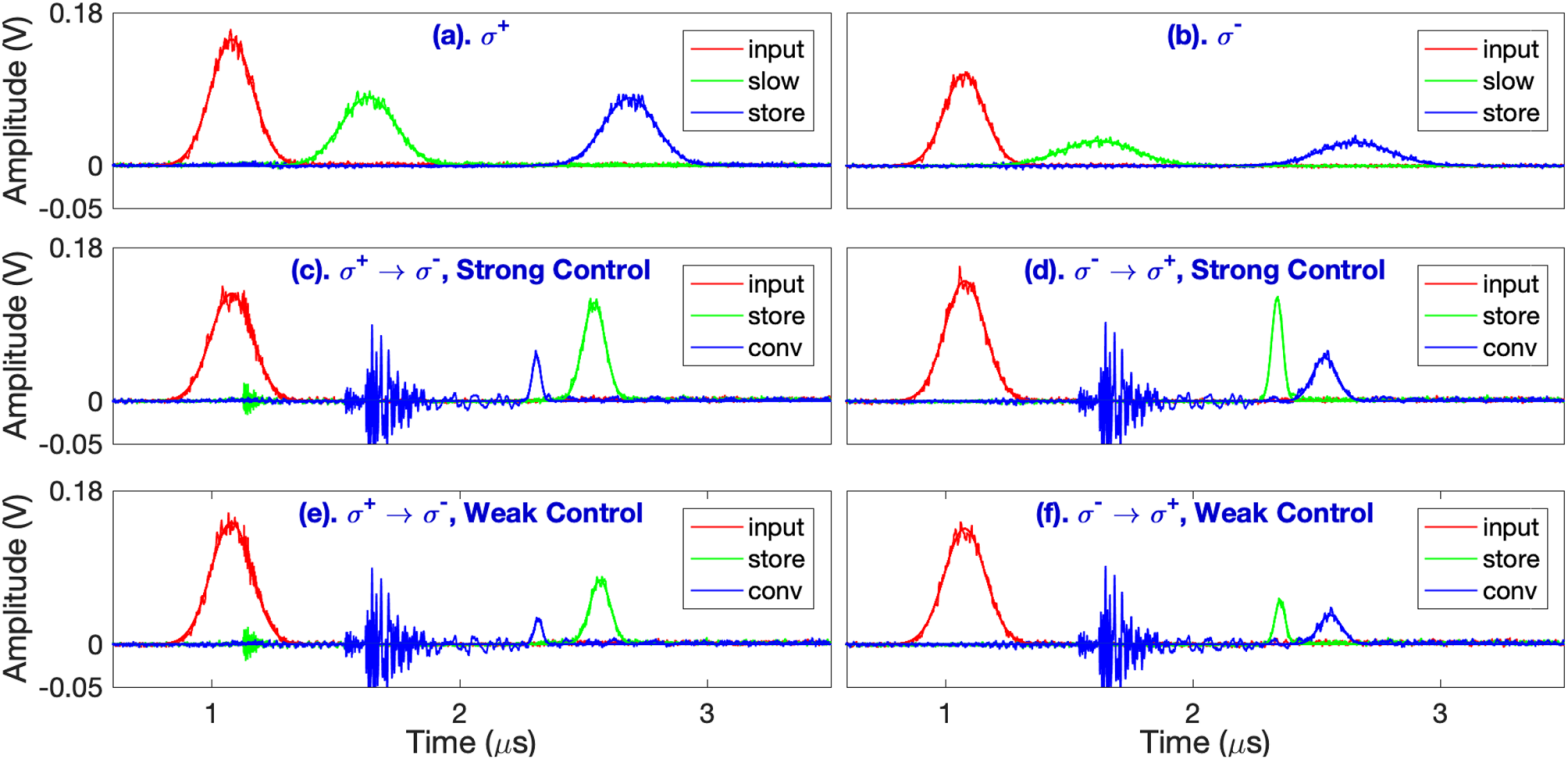
Representative raw data: (**a**) and (**b**) depict the input (red), slowed (green), and stored and retrieved (blue) probe pulses in the same EIT channel: the $\sigma ^+$ and $\sigma ^-$ channel, respectively. (**c**,**e**) and (**d**,**f**) depict the input (red), stored and retrieved pulses in the original channel (green), and the converted (blue) pulses in the $\sigma ^+ \rightarrow \sigma ^-$ and $\sigma ^- \rightarrow \sigma ^+$ conversion system, respectively. The intensities of the writing control beams for (**c**) and (**d**) are stronger than those of (**e**) and (**f**), respectively. The Rabi frequencies for the writing control beam ($\Omega _w$) are (**a**) $4.03\Gamma$, (**b**) $1.97\Gamma$, (**c**) $3.11\Gamma$, (**d**) $1.51\Gamma$, (**e**) $2.11\Gamma$, and (**f**) 0.93 $\Gamma$, respectively. The relative conversion efficiencies ($\xi _c^R$) are (**c**) $17.4\%$, (**d**) $93.8\%$, (**e**) $15.4\%$, and (**f**) $150.5\%$, respectively. The high frequency noises appearing in (**c**–**f**) at roughly $1.7\,\mu \hbox {s}$ come from the electronic noise due to the switching-on of the high voltage power supply for the electro-optic modulator. The optical depths for the $\sigma ^+$ and $\sigma ^-$ EIT system are 389(41) and 52(3), respectively, where the quantity in brackets is the $2\sigma$ standard deviation.

The experimental setup is shown in Fig. 1c. The probe beam passes twice through one acoustic-optic modulator (AOM) for tuning its frequency and then passes through another AOM for shaping its temporal waveform into a Gaussian pulse (both AOMs are not shown). In the pulsed probe experiment, the intensity full width at half maximum (FWHM) of the Gaussian probe pulse (denoted as $T_p$) is set to 200 ns. We also conduct the storage and retrieval experiment with a continuous-wave (CW) probe for a comparison with the pulsed probe case. More details of the CW probe experiment are described in the Methods section. The probe beam is sent into the MOT cell via a polarization-maintaining fiber. The control beam is passed through an electro-optic modulator (EOM) to quickly change its polarization within $\sim 10\, \hbox {ns}$ after the storage and prior to the retrieval process. The probe beam is then coupled together with the control beam through a beam splitter before entering the atomic clouds. The probe beam is focused to an intensity $e^{-2}$ diameter of $\sim 100\mu \hbox {m}$ around the atomic clouds while the control beam is collimated with a diameter of $\sim 1 \,\hbox {mm}$. After coming out of the MOT cell, both beams pass through another lens and the control beam is focused while the probe beam is collimated. The control beam is blocked by a window with a black dot in the focal plane. The probe beam passes through three irises and an etalon filter, before being coupled into a fiber and detected by a photomultiplier tube (Hamamatsu R636-10).

Figure 1d depicts the timing sequence for the pulsed experiment of polarization conversion. Detailed control to implement the dark and compressed MOT to increase the optical depth of the atomic sample are also shown. To control the ground-state coherence mismatch factor ($$\xi _2$$), which is sensitive to the Zeeman population distribution, we apply an optical pumping beam which drives the $$D_2$$ line $$|F=3\rangle \rightarrow |F'=2\rangle$$$$\sigma ^+$$ transitions^17^. It pumps the atomic population toward the Zeeman states with a larger magnetic quantum number *m*. We can control the Zeeman population distribution by controlling the duration and/or intensity of the optical pumping beam. The Zeeman population can be determined by the microwave spectroscopy, described in the Methods section. The writing control field is turned on for $$10\,\mu \hbox {s}$$ and is then turned off to store the probe pulse into spin wave. After a storage time of $$\sim 1 \,\mu \hbox {s}$$, the reading control field is turned on to retrieve the probe pulse, either in the original $$\Lambda$$ or the converted $$\Lambda$$ system.

### Experimental observations

The representative raw data are illustrated in Fig. 3. In all these measurements, the optical pumping beam is applied for $20 \mu s$ to pump atoms towards the $|F=3, m=3\rangle$ state. Figure 3a, b depict the input, slowed, and stored- and- retrieved optical pulses in the same $\Lambda$ system ($\sigma ^+$ and $\sigma ^-$, respectively). In order to achieve an optimized retrieval efficiency, we adjust the power of the control beam such that $\eta =T_d/T_p \approx 2.7$^17^. Since the optical depth of the atomic clouds corresponding to the $\sigma ^+$ channel is larger than that of the $\sigma ^-$ channel under the optical pumping condition, the measured efficiencies of the slow light (67 %) and stored light (66 %) in the $\sigma ^+$ EIT channel are larger than those (49 %, 45 %) of the $\sigma ^-$ EIT channel^17^.

**Figure 4:**
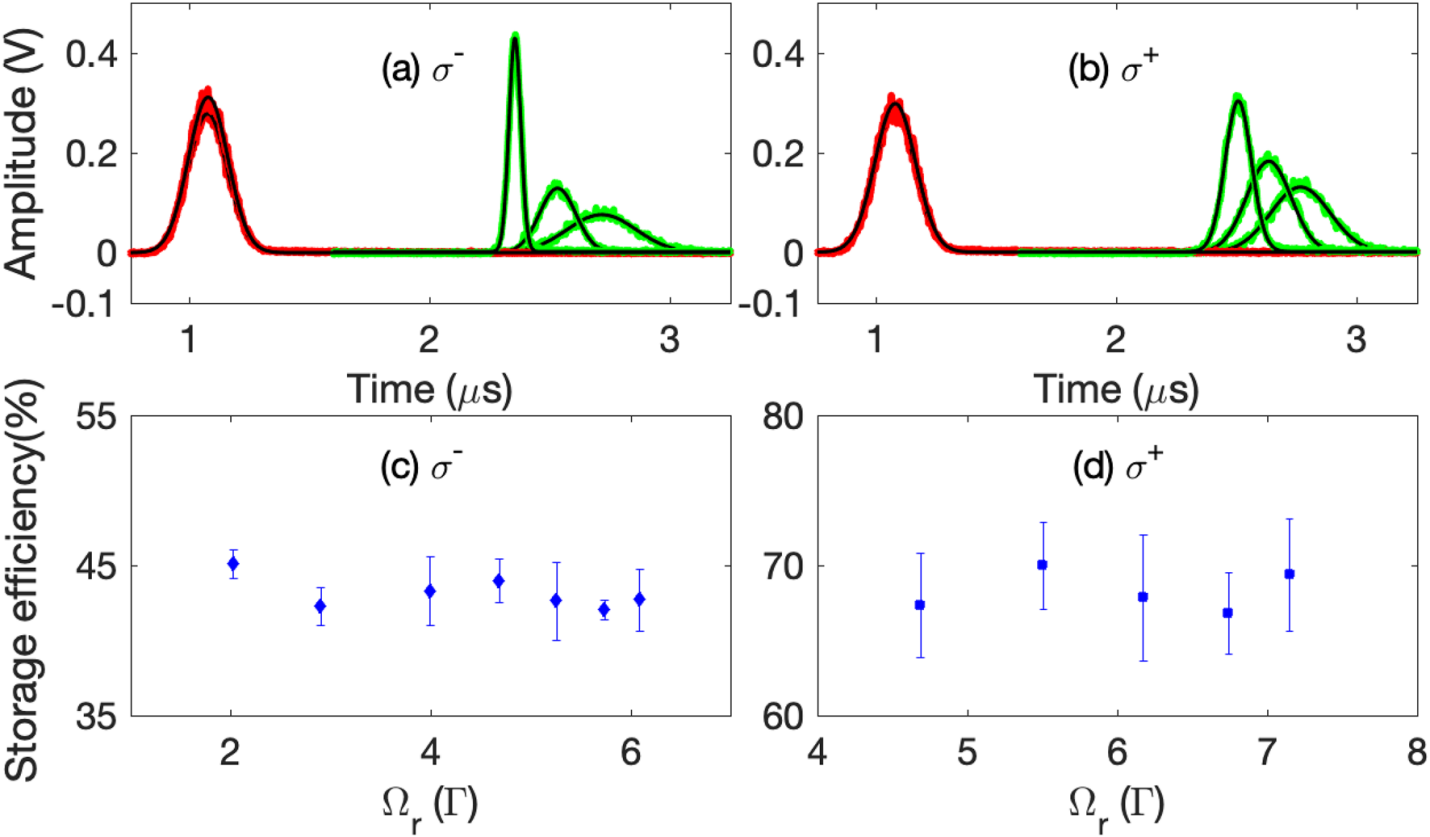
(**a**) and (**b**) depict representative data of the stored and retrieved pulses (green) for some intensities of the reading control beam in the $\sigma ^+$ and $\sigma ^-$ channel, respectively. The red trace is the input pulse. All black solid lines are the Gaussian fits to the data. In (**a**), the Rabi frequencies of the reading control beam ($\Omega _r$) for the three retrieval cases are 2.02, 2.90 and 6.09 $\Gamma$. In (**b**), the $\Omega _r$ are 4.68, 5.50, and 6.74 $\Gamma$. (**c**) and (**d**) depict the storage efficiency versus $\Omega _r$ for the $\sigma ^-$ and $\sigma ^+$ channel, respectively. The optical depth of the $\sigma ^+$ and $\sigma ^-$ EIT system are 349(32) and 47(3), respectively. Error bars in (**c**) and (**d**) are the statistical standard deviation of many data runs taken at the same experimental conditions.

Below, we present the data related to polarization conversion. Figure 3c,e (Fig. 3d,f) correspond to the case of storing via the $\sigma ^+$ channel ($\sigma ^-$ channel) and retrieving via the $\sigma ^-$ channel ($\sigma ^+$ channel). Both Fig. 3c, d depict the data with a stronger writing control field, compared to that of Fig. 3e, f in which a weaker writing control field was used. The finite bandwidth factor $\xi _1$ should be less than unity in the $\sigma ^+ \rightarrow \sigma ^-$ conversion case, since the conversion is from the channel with a larger optical depth into that with a smaller one. Also, the coherence mismatch factor $\xi _2$ for this conversion case is smaller than unity because the Zeeman population is not completely in a single Zeeman state and there exist a Clebsch–Gordan coefficient mismatch between the two $\Lambda$ systems. Thus, based on Eq. (1), the relative conversion efficiency $\xi _c^R$ is smaller than unity, which agrees with both Fig. 3c, e. In the case shown in Fig. 3e with a weaker writing control field and thus a larger $\eta$, $\xi _1$ decreases further because of a stronger finite bandwidth effect which leads to an even smaller $\xi _c^R$. In the opposite case of $\sigma ^- \rightarrow \sigma ^+$ conversion, the $\xi _1$ factor is larger than unity since it is converted from a system with a smaller optical depth into that with a larger one. However, the $\xi _2$ factor is still smaller than unity and these two factors compete against each other. In the case with a stronger writing control regime (and thus a smaller $\eta$), the finite bandwidth effect is relatively less significant and $\xi ^R_c$ is mainly affected by the coherence mismatch factor $\xi _2$. Thus, the overall $\xi ^R_c$ is still smaller than unity (93.8%), corresponding to the case of Fig. 3d. In the case with a weaker writing control beam (and thus a larger $\eta$), the finite bandwidth effect dominates and $\xi _1$ could be larger than unity. Therefore, the overall $\xi ^R_c$ could be larger than unity for a large enough $\eta$, such as that of Fig. 3f which has a relative conversion efficiency of 150.5%. In this case, the efficiency of the converted light is even larger than that retrieved in the original channel. Although the relative conversion efficiency $\xi _c^R$ could be larger than unity, the absolute conversion efficiency is significantly lower than unity since $\xi _c^R >1$ occurs at a relatively large $\eta$, in which the efficiency of the storage process is relatively low.

It should be noted that the pulse height and temporal width of the retrieval signal can be manipulated by the intensity of the reading control beam (with its Rabi frequency denoted as $\Omega _r$)^4,5^. In an ideal three-level $\Lambda$-type EIT system, the retrieval efficiency should be independent of $\Omega _r$^15^. However, in real atoms, nearby transitions may exist such that the off-resonant excitation of the control beam may induce a $\Omega _r$-dependent multi-photon decay of the spin-wave^17,19^. Fortunately, the nearest off-resonant excitation of the control field for the cesium $D_1$ line is quite far-detuned ($\sim 1.167 \,\hbox {GHz}$ for the $|6S_{1/2}, F=4\rangle \rightarrow |6P_{1/2}, F=3\rangle$ transition) such that the decay of the spin-wave due to the aforementioned mechanism is negligible, at least for low $\Omega _r$^17^. To check if this is true, we measure the retrieval efficiency with different $\Omega _r$ for both the $\sigma ^-$ and $\sigma ^+$ EIT systems, as shown in Fig. 4. Within the experimental uncertainty, the retrieval efficiencies are roughly constant for the range of $\Omega _r$ used. This trend may not be valid if one implement the EIT-memory-based converter using the $D_2$ transition^17^. Because of this fact and in order to make the retrieval signal more clearer, we choose a relatively strong reading control field to retrieve the signal, such as those shown in Fig. 3.

**Figure 5:**
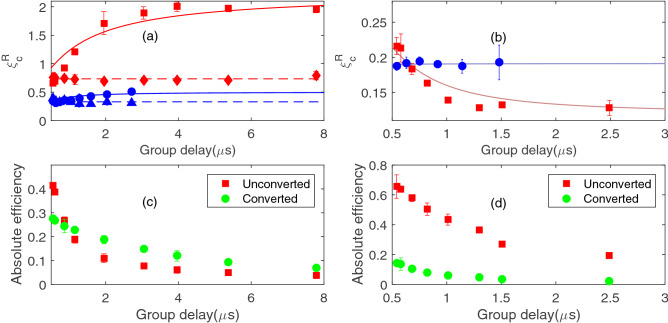
Relative conversion efficiency $\xi ^R_c$ versus group delay time of the storage process for the (**a**) $\sigma ^- \rightarrow \sigma ^+$ and (**b**) $\sigma ^+ \rightarrow \sigma ^-$ conversion systems. In (**a**), the red squares (diamonds) data correspond to the pulsed (continuous) probe data with Zeeman optical pumping which pumps the population towards the $|m=3\rangle$ state. The blue circles (triangles) data correspond to the pulsed (continuous) probe data for the case without Zeeman optical pumping. The two dashed lines correspond to the average values of $\xi ^R_c$ for the continuous case. The two solid curves relate to the calculation by Eq. (1) with $\alpha _p=\alpha _c=500$, $\kappa =1$, and the population from $|F=3, m=3\rangle$ to $|F=3,m=-3\rangle$ states to be (0.60, 0.24, 0.14, 0.02, 0, 0, 0) and (0.25, 0.25, 0.15, 0.15, 0.10, 0.05, 0.05) for the case with and without Zeeman optical pumping, respectively. In (**b**), the red squares (blue circles) correspond to the pulsed probe data with and without optical pumping. The population from m = 3 to −3 states are (0.52 0.24, 0.08, 0.04, 0.03, 0.02, 0.07) and (0.19, 0.17, 0.11, 0.10, 0.07, 0.17, 0.20), respectively. The two solid lines are related to the calculation by Eq. (1) with $\kappa =1$ and $\alpha _p=\alpha _c=500$. In (**c**) and (**d**), the absolute efficiency for the unconverted (red square) and converted (green circle) case for the data with optical pumping in (**a**) and (**b**), respectively. Error bars are the statistical standard deviation of many data runs taken at the same experimental conditions.

To gain a more comprehensive picture, we conduct systematic measurements of $\xi ^R_c$ versus $\eta$ by varying the $\Omega _w$ for various configurations. In the measurements, we keep $T_p$= 200 ns. We consider both the $\sigma ^- \rightarrow \sigma ^+$ and $\sigma ^+ \rightarrow \sigma ^-$ conversion systems, which correspond to storage in a system with smaller optical depths and retrieval in another system with larger optical depths and vice versa. In each system, we also consider varying the Zeeman population distribution by applying an optical pumping beam. To explore the finite bandwidth effect, we also consider storage and retrieval with a continuous-wave (CW) probe, to serve as a reference for the pulsed probe case.

Figure 5a depicts the results for the $\sigma ^- \rightarrow \sigma ^+$ conversion system. The red squares and blue circles indicate the data for the pulsed probe case with Zeeman optical pumping beam turned on and off, respectively. The red diamonds and blue triangles indicate the data for the CW probe. It is evident that $\xi _c^R$ for the CW case shows almost no dependence on the group delay time $T_d$. This is not surprising since the finite-bandwidth effect in the CW case is negligible and $\xi ^R_c$ is only determined by $\xi _2$. Without optical pumping, the Zeeman population is nearly isotropic and the $\xi _c^R$ shows a very weak dependence on $\eta$. With optical pumping, the Zeeman population concentrated towards Zeeman states with a larger *m* and $\xi _c^R$ increases with $\eta$. At a large enough $T_d$, $\xi _c^R$ is larger than unity, approaching to a maximum value of $\sim$2 at large $T_d$. For the CW probe case, $\xi _c^R$ is larger for the case with optical pumping because the Zeeman population is more concentrated, which favors a larger $\xi _2$. As a reference, we also plot the absolute efficiency for the unconverted and converted case, as shown in Fig. 5c for the data in Fig. 5a with optical pumping. At a large enough group delay, the absolute efficiency for the converted case surpasses that of the unconverted case, which leads to $\xi _c^R>1$ for those data shown in Fig. 5a. All these behaviors are similar to the theoretical predictions illustrated in Fig. 2.

The results of the corresponding experiments conducted on the $\sigma ^+ \rightarrow \sigma ^-$ conversion system are shown in Fig. 5b. Here, opposite to the previous case, the light is converted from the system with a large optical depth to that with a small one. The finite bandwidth factor $\xi _1$ is thus less than unity, which results in a decrease of $\xi ^R_c$ when $T_d$ increases. This behavior is more significant when the optical pumping is on and the Zeeman population concentrated towards the Zeeman states with a larger *m*, which leads to a smaller optical depth for the conversion transition. In Fig. 5d, we plot the absolute efficiency for the unconverted and converted case for the data in 5b with optical pumping. It is evident that the absolute efficiencies for the converted case are all less than those of the unconverted case. This behavior is very different from the $\sigma ^- \rightarrow \sigma ^+$ conversion system. Without the optical pumping, $\xi ^R_c$ is insensitive to $T_d$ because the Zeeman population is more close to an isotropic distribution and the finite-bandwidth factor $\xi _1$ is near unity, as shown in the blue circle data of Fig. 5b. The observed behaviors are similar to the theoretical predictions.

## Conclusion

We conduct an experiment demonstrating EIT-memory-based optical polarization conversion in a double-$\Lambda$ system with cold cesium atoms. The dependence of the conversion efficiency on the degenerate Zeeman states and finite bandwidth effect is studied. The experimental observations support the theoretical predictions. Our studies provide essential knowledge for the practical implementation of EIT-memory-based optical converters.

## Methods

### Measurement of the continuous-wave (CW) conversion efficiency

**Figure 6:**
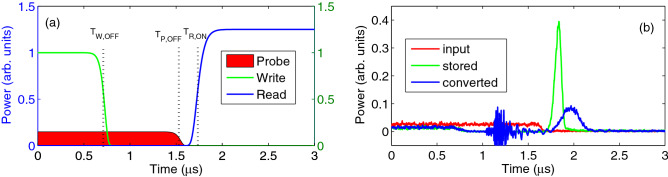
(**a**) Schematic timing diagram for the probe (red), writing control (green) and reading control (blue) fields for implementation of the continuous probe storage and conversion measurement. The reading control beam could be that of the original $\Lambda$ channel or the converted $\Lambda$ channel. (**b**) Representative data for the input continuous probe (red), the retrieved probe fields in the original (green) and in the converted (blue) $\Lambda$ channel. The noise around $1.7\,\mu \hbox {s}$ is due to the switching on of the high-voltage driver for the EOM.

To implement the storage and conversion of the continuous-wave (CW) probe field, we first turn on both the writing control beam and the probe beam. The probe power is kept at a constant value. The writing control beam is switched off at a given time (denoted as $T_{W,OFF}$). After a specified time, the probe beam is also turned off at time denoted as $T_{P,OFF}$. This ensures that the atomic ensemble is filled by a certain portion of the probe beam of constant intensity such that its behavior is more like the CW case. At time $T_{W,OFF}$, the portion of the probe beam that enters the atomic medium will be converted to and stored as a spin-wave. At a time (denoted as $T_{R,ON}$) later than $T_{P,OFF}$, the writing control beam, either of the original $\Lambda$ system or the converted one, is turned on to retrieve the probe pulse in the original or in the converted $\Lambda$ system, respectively. The schematic timing diagram is shown in Fig. 6a. One set of representative data is shown in Fig. 6b. The ratio of the retrieved probe energy in the converted $\Lambda$ system (blue) to that in the original $\Lambda$ system (green) is the relative conversion efficiency of the CW case.

**Figure 7:**
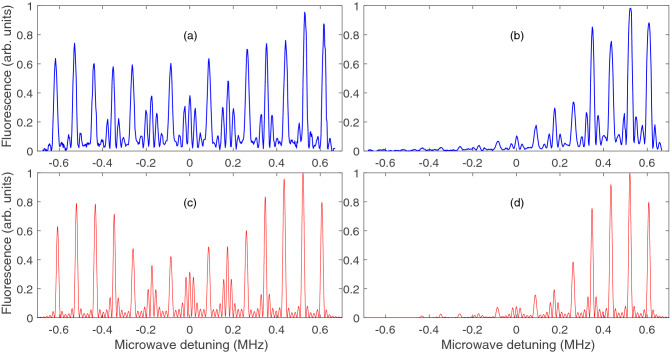
(**a**) and (**b**) are the representative microwave spectrum with Zeeman optical pumping off and on, respectively. The x-axis is the microwave frequency minus 9192.63177 MHz, the hyperfine splitting between the $|F=3\rangle$ and $|F=4\rangle$ ground state. (**c**) and (**d**) are the calculated microwave spectrum corresponding to (**a**) and (**b**) with population from $|m=3\rangle$ to $|m=-3\rangle$ to be (0.15, 0.16, 0.16, 0.14, 0.13, 0.13, 0.13) and (0.38, 0.38, 0.15, 0.06, 0.02, 0.01, 0), respectively. In the calculation, the magnetic field is 248 mG. The microwave Rabi frequencies for the $\pi$ transitions are $3.936\times 10^5\,\hbox {Hz}$, multiplied by the Clebsch–Gordon coefficients. The Rabi frequency ratio for the microwave $\sigma$ to $\pi$ transition is 0.36, which is related to the angle between the microwave magnetic field to the applied DC magnetic field.

### Determination of the Zeeman population by microwave spectroscopy

Microwave spectroscopy is conducted to determine the Zeeman population distribution. During measurement, a magnetic field of $\approx 248 mG$ is applied and a microwave pulse of $50\,\mu \hbox {s}$ duration is turned on through a horn antenna to drive atoms from the $|6S_{1/2}, F=3\rangle$ Zeeman states into the $|6S_{1/2}, F=4\rangle$ Zeeman states. The trapping beams are then turned on with the frequency jumping to the resonance of the $|6S_{1/2}, F=4\rangle \rightarrow |6P_{3/2}, F'=5\rangle$ cycling transition. One CCD camera is then used to collect the atomic fluorescence for $70\,\mu \hbox {s}$. The microwave frequency is scanned through 9.192 GHz and the timing sequence repeated to get the spectrum. It can be seen that 15 major lines appear in the spectrum due to the Zeeman shifts in the example shown in Fig. 7a and b with the Zeeman optical pumping off and on, respectively. Some lines are split into several sub-lines due to the relatively strong microwave field used in the experiment. Because the total trapping laser intensity is very strong ($\sim 100 \,\hbox {mW}/\hbox {cm}^2$ corresponding to an on-resonance saturation parameter of $\sim$37 with respect to the isotropic saturation intensity of $2.706 \,\hbox {mW}/\hbox {cm}^2$)^20^, the fluorescence rate for each Zeeman sublevel is almost saturated to $\Gamma /2$ and the relative intensities of the spectral peaks are determined by the Zeeman population and the relative strength of the microwave transitions only. By varying the parameters including the microwave Rabi frequency, the Rabi frequency ratio between the microwave $\pi$ to $\sigma$ transition and the Zeeman population, the calculated microwave spectrum closely resembles the experimental spectrum, as shown in Fig. 7c, d. As can be seen in Fig. 7d, the $\sigma ^+$ optical pumping beam pumps the Zeeman population towards the Zeeman states with a larger magnetic quantum number *m*.
